# Stabilization of Expansive Soils Using Cement–Zeolite Mixtures: Experimental Study and Lasso Modeling

**DOI:** 10.3390/ma18102286

**Published:** 2025-05-14

**Authors:** Ibrahim Haruna Umar, Sale Abubakar, Abdullahi Balarabe Bello, Hang Lin, Jubril Izge Hassan, Rihong Cao

**Affiliations:** 1 School of Resources and Safety Engineering, Central South University, Changsha 410083, China; ibrahimharunaumar@yahoo.com; 2 Department of Civil Engineering, Faculty of Engineering, Aliko Dangote University of Science and Technology, Wudil 713101, Nigeria; salehabubakarrr@gmail.com; 3 Department of Civil Engineering, Bayero University, Kano 700241, Nigeria; bbello2015@gmail.com; 4 Department of Geology, Faculty of Physical Sciences, Ahmadu Bello University Zaria, Zaria 810211, Nigeria; jihassan@abu.edu.ng

**Keywords:** expansive soil stabilization, cement–zeolite synergy, swell and shrinkage, California bearing ratio (CBR), principal component lasso regression (PCLR), mediation, moderation, conditional process analysis using PCLR model

## Abstract

The stabilization of expansive soils is crucial for the construction projects to mitigate swelling, shrinkage, and bearing capacity issues. This study investigates the synergistic effects of cement and clinoptilolite zeolite on stabilizing high-plasticity clay (CH) soil from Kano State, Nigeria. A total of 30 admixture combinations—cement (0–8%) and zeolite (0–15%)—were tested via standardized laboratory methods to evaluate their free swell index (FSI), swell percentage, swell pressure, shrinkage, and California Bearing Ratio (CBR). Principal component (Lasso) “least absolute shrinkage and selection operator” regression modeled interactions between admixtures and soil properties. The key results include the following: (1) 6% cement + 12% zeolite reduced the FSI by 60% (45 → 18); (2) 8% cement + 15% zeolite decreased the swell percentage by 47.8% (22.5% → 11.75%); (3) 6% cement + 12% zeolite lowered swell pressure by 54.2% (240 kPa → 110 kPa); (4) 8% cement + 12% zeolite reduced shrinkage by 50% (5.6% → 2.8%); and (5) 6% cement + 9% zeolite achieved an unsoaked CBR of 80.01% and soaked CBR of 72.79% (resilience ratio: 0.8010). PCLR models explained 93.5% (unsoaked) and 75.0% (soaked) of the CBR variance, highlighting how zeolite’s mediation analysis indicates that zeolite improves the bearing capacity mainly by reducing the free swell index (path coefficient = −0.91429, *p* < 0.0001), while conditional process modeling provided greater explanatory power (R^2^ = 0.745) compared to moderation-only analysis (R^2^ = 0.618). This study demonstrates that zeolite–cement blends optimize strength and resilience in expansive soils, with implications for sustainable infrastructure in arid and semi-arid regions.

## 1. Introduction

Expansive soils, predominantly composed of clay minerals like montmorillonite, pose significant challenges in geotechnical engineering and construction due to their tendency to undergo substantial volume changes with variations in moisture content [1]. When these soils become saturated, they can swell considerably, exerting enormous swell pressures that can heave and damage overlying structures such as buildings, roads, and pipelines [2]. Conversely, expansive soils can shrink during dry periods, leading to ground settlement and cracking issues. These cyclic swell–shrink behaviors jeopardize infrastructure’s integrity and increase maintenance costs over the service life of facilities constructed on expansive soil deposits [3]. Conventional methods to mitigate expansive soil problems involve techniques like soil replacement, moisture control, compaction control, and the provision of adequate flexible foundations [4]. However, these approaches can be labor-intensive, time-consuming, and expensive, especially for large-scale projects. As a result, soil stabilization using admixtures has emerged as a cost-effective and sustainable alternative to improve the engineering properties of problematic soils [5]. Soil stabilization represents a critical aspect of civil engineering practice, allowing for the improvement of inadequate soil properties through physical and chemical modifications. Among various stabilizing agents, cement has maintained its prominence as a traditional binder, while zeolites have emerged as sustainable alternatives due to their unique pozzolanic properties and environmental benefits [6]. The complexity of soil–binder interactions and the multifaceted nature of stabilized soil behavior, particularly under dynamic loading conditions, present significant challenges for conventional analytical approaches. This complexity has catalyzed the adoption of artificial intelligence methodologies to model, predict, and optimize the properties and performance of cement and zeolite-stabilized soils.

Cement has been widely used as a stabilizing agent due to its ability to increase the strength and reduce the swell–shrink potential of expansive soils through cation exchange, flocculation, and pozzolanic reactions [7]. However, using cement alone may not always be sufficient or economical, mainly when dealing with highly expansive soils or regions with limited cement availability [8,9]. Recently, there has been a growing interest in supplementing cement with pozzolanic materials, such as fly ash, blast furnace slag, and natural pozzolans like zeolites, to enhance the stabilization process and improve the overall performance [10]. Zeolites are naturally occurring aluminosilicate minerals with a highly porous and crystalline structure. They possess unique properties, including a high cation exchange capacity, pozzolanic reactivity, and water adsorption/desorption capabilities, making them attractive for soil stabilization applications [11]. Zeolites can participate in pozzolanic reactions when combined with cement, forming additional cementitious compounds that contribute to the development of strength and reduced permeability [6]. Furthermore, their water retention and controlled release characteristics can potentially mitigate shrinkage issues in stabilized soils during drying cycles [12].

The prediction and characterization of dynamic mechanical properties represent particularly challenging aspects of stabilized soil analysis, due to the complex dependency on loading conditions, frequency, stress history, and materials’ evolution. Advanced artificial intelligence (AI) models have demonstrated remarkable capabilities in capturing these multifaceted relationships across diverse stabilization scenarios [13]. Despite significant advances, several challenges persist in the application of AI for cement and zeolite soil stabilization [12]. Data scarcity represents a fundamental limitation, as comprehensive datasets covering diverse soil types, stabilizer combinations, and loading conditions remain rare. This scarcity is particularly acute for zeolite-based stabilization, where standardized testing protocols and systematic property characterization are still evolving. The quantification of predictions’ uncertainty represents a critical but often neglected aspect of AI implementation in geotechnical engineering [11]. Deterministic predictions without associated confidence intervals provide incomplete information for risk-based design decisions [14]. While Bayesian neural networks and ensemble methods offer theoretical frameworks for uncertainty quantification, their practical implementation in soil stabilization applications remains limited [13]. The integration of mediation, moderation, and conditional process analysis into studies of cement–zeolite soil stabilization, particularly when modeling dynamic mechanical properties (e.g., seismic loading), represents a paradigm shift in geotechnical engineering. This approach, combined with advanced AI-driven hybrid models, addresses critical gaps in understanding complex soil-additive interactions and their implications for infrastructure’s resilience.

Soil stabilization using cement and zeolite has emerged as a sustainable solution for enhancing geotechnical properties, particularly in problematic active regions. Recent studies emphasize the synergistic effects of these additives, coupled with advanced modeling techniques to predict dynamic mechanical behavior under earthquake loading [11]. Cement and zeolite combinations are critical for seismic resilience; experimental studies demonstrate that replacing 30% of cement with zeolite increases the unconfined compressive strength (UCS) by 15–25% [15], attributed to zeolite’s pozzolanic activity and ion-exchange capacity [16]. Recent advances in fractional-order viscoelastic models and constitutive equations simulate the strain-rate dependency and damping ratios in stabilized soils. For example, Molaabasi and Shooshpasha [17] developed a soil–cement model for shear strains ranging from 10^−6^ to 10^−2^, critical for predicting liquefaction resistance. Shang et al. [18] proposed a damage mechanism for frozen moraine soils under impact loading, offering insights into dynamic modulus degradation. Despite these advances, few studies integrate dynamic properties with additive-induced microstructural changes, limiting their predictive scalability. AI and hybrid models address nonlinear relationships in soil-additive systems. Nasrollahpour et al. [18] used multi-objective optimization through hybrid models, combining genetic algorithms with finite-element analysis (FEA) to optimize cement–zeolite dosages while minimizing environmental impacts. Turkoz and Vural [18] quantified uncertainty, using probabilistic ML models to improve the reliability in predicting dynamic properties (e.g., cyclic resistance) under variable field conditions. However, the integration of AI remains underexplored for consolidation and CBR modeling, particularly in linking micro-scale mediators (e.g., pore structure) to macro-scale outcomes.

Hybrid ensemble models have shown promising results in predicting damping behavior in cement and zeolite-stabilized soils, with Nguyen et al. [19] combining gradient boosting machines with genetic algorithm feature selection to achieve correlation coefficients above 0.92, while specialized LSTM and GRU recurrent neural networks have demonstrated exceptional performance in modeling the cyclic response, as seen in the work of Zhou, Tian [19] capturing stiffness degradation and the attention-augmented architectures of Xie, Qiu [20] for zeolite-blended systems. Sensor networks embedded within stabilized soil layers have enabled real-time performance prediction and the detection of early degradation through transformer-based models (El-Shafeiy et al. [21]) and hybrid physics–ML frameworks (Taloma et al. [21]), though the dynamic integration of mediation analysis (e.g., the free swell index as a mediator) and moderation effects (e.g., cement–zeolite interactions) is rarely coupled with these dynamic models, creating opportunities for hybrid AI frameworks to bridge this gap by simulating how mediators influence mechanical resilience, particularly with respect to underexplored factors like swelling as moderators of dynamic behavior, despite their relevance to long-term stability [14].

This study aims to investigate the synergistic effects of combining ordinary Portland cement (0–8%) and clinoptilolite zeolite (0–15%) in stabilizing high-plasticity clay (CH) soil from Kano State, Nigeria. Specifically, it seeks to do the following: 1. Quantify the impact of cement–zeolite blends on critical geotechnical properties, including the free swell index (FSI), swell percentage, swell pressure, shrinkage coefficient, and CBR under unsoaked and soaked conditions. 2. Determine optimal admixture ratios that minimize swelling–shrinkage while maximizing strength and resilience, using a systematic matrix of 30 cement–zeolite combinations. 3. Employ principal component Lasso regression (PCLR) to model complex interactions between admixtures and soil properties, identifying mediation effects (e.g., FSI as a mediator of improvements in strength) and optimizing the mixture’s design for field applications. This study addresses gaps in sustainable soil stabilization by integrating mechanistic insights (cation exchange, pozzolanic reactions) with advanced statistical modeling to advance cost-effective, environmentally friendly solutions for infrastructure in expansive soil regions.

## 2. Materials and Methods

### 2.1. Soil Sample and Materials

**Figure 1:**
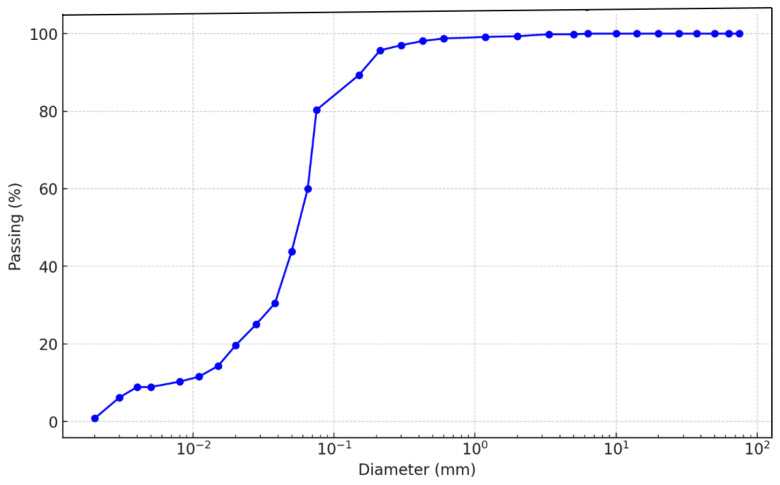
Particle size distribution curve of the soil.

The soil used in this study was obtained from a depth of 2–3 m at a construction site in Dantsinke, Kano State, Nigeria, known for the prevalence of expansive soils. The soil was classified as high-plasticity clay (CH) according to the Unified Soil Classification System (USCS), with a liquid limit of 68% and a plasticity index of 49%. The particle size distribution (PSD) curve in Figure 1 was determined through a combination of sieve analysis for the coarse fraction (gravel and sand) and hydrometer analysis for the fine-grained fraction (silt and clay). These methods are standard for soils with a high fines content. The testing protocol followed ASTM guidelines rigorously, including oven-drying, the disaggregation of soil clumps, and dispersion using sodium hexametaphosphate to ensure accuracy. Table 1 summarizes the material properties of soil, zeolite, and cement. These properties suggest zeolite and cement could synergistically enhance the soil’s mechanical and chemical stability. The specific gravity of soil solids was determined to be 2.7 following ASTM D854-14 [21]. The cement used was ordinary Portland cement obtained from Dangote Cement PLC (Kogi, Nigeria). At the same time, the zeolite was a natural clinoptilolite zeolite obtained from a mining operation and ground to a fine powder passing a No. 200 sieve.

The natural soil’s high plasticity (LL = 68%, PI = 49%) and swelling–shrinkage tendencies are attributed to its dominant montmorillonite clay mineralogy. Montmorillonite’s expansive nature arises from its 2:1 smectite structure, characterized by a high cation exchange capacity (CEC) and interlayer adsorption of water molecules [22]. The soil’s acidic pH (5.12) and low calcium content (inherent in montmorillonite-rich systems) further exacerbate its instability, as calcium-deficient clays exhibit weaker flocculated structures compared to Ca^2+^-stabilized systems [23]. Zeolite and cement were selected as stabilizers to address these deficiencies: zeolite introduces a high CEC (1.1 meq/g) for ion exchange, while cement provides calcium ions (Ca^2+^) to promote pozzolanic reactions and clay particle aggregation [24].

Both cement and zeolite samples were homogenized by grinding them to a fine powder (<75 µm particle size) using a planetary ball mill. Major oxides (SiO_2_, Al_2_O_3_, Fe_2_O_3_, CaO, etc.) were quantified using wavelength-dispersive X-ray fluorescence (XRF) spectroscopy (PANalytical Axios MAX). Cement samples were fused with lithium borate flux at 1050 °C to form glass beads, while zeolite powders were pressed into pellets with a binder. XRF is widely recognized for its precision in analyzing cementitious and aluminosilicate materials [25,26]. For improved accuracy in detecting light elements (Na_2_O, MgO), calibration curves were validated using certified reference materials (NIST SRM 1880a for cement and NIST SRM 2697 for zeolites). Inductively Coupled Plasma Optical Emission Spectrometry (ICP-OES; PerkinElmer Avio 500) was employed after acid digestion (HNO_3_/HF mixture for zeolites; HNO_3_/HCl for cement). Detection limits for trace constituents (e.g., Cl, PbO) were established via blank corrections. Loss on ignition (LOI) was measured by heating samples in a muffle furnace (1 g sample, 950 °C for cement; 550 °C for zeolite to preserve its framework integrity) until constant mass was achieved, following ASTM C114 [27] for cement and a modified protocol for zeolite [28]. Triplicate analyses were performed, and the results were cross-validated with internal standards. The uncertainties were <2% for major oxides and <5% for trace elements.

The chemical composition data presented in Table 2 reveal fundamental differences between zeolite and cement that explain their complementary stabilization mechanisms. Zeolite exhibits a predominantly siliceous character (58.9% SiO_2_) with significant alumina (11.3% Al_2_O_3_), forming the characteristic aluminosilicate framework of clinoptilolite. Its notable alkali content (Na_2_O: 3.8%, K_2_O: 3.7%) indicates a substantial cation exchange capacity, enabling an interaction with clay minerals through cation replacement, potentially reducing soil’s expansiveness. Conversely, cement is dominated by calcium oxide (62.62% CaO), responsible for its cementitious properties through the formation of calcium silicate and aluminate hydrate. The considerable difference in loss on ignition values (zeolite: 19.03%, cement: 2.38%) reflects zeolite’s microporous nature and capacity to retain water molecules within its crystalline structure, contributing to moisture regulation in stabilized soil systems. These distinct chemical profiles elucidate their complementary roles when used in combination: cement provides direct chemical binding and the immediate development of strength, while zeolite’s siliceous nature and ion-exchange properties enhance long-term durability and environmental resistance.

### 2.2. Experimental Procedures and Protocols

#### 2.2.1. Specimen Preparation

This study evaluated soil mixtures incorporating varying percentages of zeolite (0–15%) and cement (0–8%), with the soil content adjusted proportionally (77–100%). All 30 combinations underwent standardized testing at approximately 22 °C, including the assessment of physical properties, swell/collapse behavior, shrinkage using the wax method, and CBR. Each increment of zeolite (0, 3, 6, 9, 12, 15%) was systematically tested against cement additions (0, 2, 4, 6, 8%), maintaining triplicate samples to ensure statistical reliability. Specimen preparation followed a rigorous standardized procedure. Natural soil was air-dried and pulverized to eliminate clods, then sieved through a No. 4 sieve (4.75 mm opening) to ensure a consistent maximum particle size. For each specimen, predetermined amounts of cement and zeolite were measured by dry weight and thoroughly mixed with the soil using a mechanical mixer to achieve homogeneity. The dry soil–admixture combinations were transferred to separate containers, where distilled water was added to achieve an optimal moisture content, as determined by standard Proctor compaction test results (ASTM D698-12) [29]. After ensuring a uniform moisture distribution, the moistened soil–admixture blends were compacted in standard Proctor molds (943 cm^3^) using a manual rammer. Compaction followed ASTM D698-12 guidelines, applying the standard Proctor effort of 600 kN-m/m^3^ in three equal layers. The compacted specimens were then extracted, trimmed to the required dimensions, and subjected to the test battery. All tests were performed in triplicate, with standard quality control measures including regular equipment calibration and strict adherence to established laboratory protocols throughout the testing program.

#### 2.2.2. Experimental Methods for Swell–Shrink and CBR Characterization

The free swell index test followed ASTM D5890-19 [30]. A portion of the compacted specimen was carefully trimmed into a cylindrical shape with a height-to-diameter ratio of 1. The specimen was then transferred to a consolidometer ring and placed in a swell dish filled with distilled water. After a curing period of 24 h, the change in the specimen’s height was measured, and the free swell index was calculated as the percentage of the initial specimen height. The swell percentage and swell pressure tests were conducted using a conventional oedometer apparatus, following the guidelines outlined in ASTM D4546-21 [31]. Compacted specimens with a diameter of 63.5 mm and a height of 20 mm were prepared and placed in the oedometer ring. After applying the desired seating pressure (typically 7 kPa), the specimens were inundated with distilled water, and the resulting swell was monitored. The swell percentage was calculated as the percentage increase in the specimens’ height upon saturation. In contrast, the swell pressure was recorded as the maximum pressure required to maintain the initial height of the specimen during swelling.

**Figure 2:**
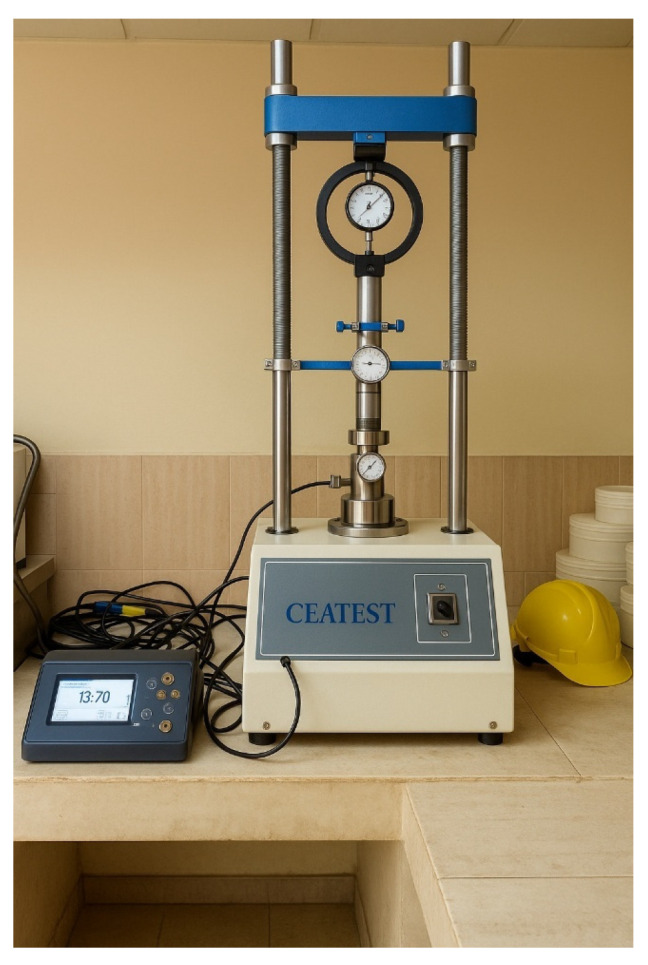
CBR machine.

The shrinkage characteristics were evaluated following ASTM D4943-18 [32]. Compacted soil specimens were carefully trimmed to form smooth bars and coated with a thin layer of microcrystalline wax. The coated specimens were then immersed in a bath of molten wax and allowed to cool, forming a wax mold that preserved the initial volume of the specimen. The specimens were then oven-dried at 105 °C until constant mass was achieved. The shrinkage percentage was calculated as the percentage decrease in the specimens’ volume from the initial to the oven-dried state. The shrinkage coefficient, which quantifies the volume change per unit change in moisture content, was also determined using the data obtained from this test. The CBR tests were performed following ASTM D1883-21 [33]. Compacted specimens with a diameter of 152.4 mm and a height of 127 mm were prepared and subjected to either unsoaked or soaked conditions. For the unsoaked CBR, the specimens were tested immediately after compaction. For the soaked CBR, the specimens were subjected to a 96 h soaking period before testing. The CBR values were determined by measuring the resistance to penetration of a standard piston at specified deformation levels and comparing the results to the resistance of a well-graded crushed stone base material. Figure 2 shows the CBR machine.

### 2.3. Statistical Methods

#### 2.3.1. Principal Component Lasso Regression (PCLR) (Version 3.9.7, Python Software Foundation, Wilmington, DE, USA)

PCLR is an advanced statistical technique that combines the dimensional reduction capabilities of principal component analysis (PCA) with the feature selection and regularization properties of Lasso regression [34]. This method is particularly useful in addressing the multicollinearity and overfitting issues often encountered in complex datasets, such as those in geotechnical engineering [35,36]. PCLR is a hybrid technique that combines PCA with Lasso regression to predict CBR values for both unsoaked and soaked conditions in soil mixtures containing varying proportions of cement and zeolite. The comprehensive analysis of the principal component Lasso regression (PCLR) method applied in this geotechnical study follows:

The PCA step begins with the standardization of the predictor variables (*X*) to ensure all variables are on the same scale from Equation (1).

(1) $$X_{std}=\frac{x-\mu }{\sigma }$$

where *μ* represents the mean, *X* is the original value, σ is the standard deviation of each variable, and $X_{std}$ is the standardized value.

The covariance matrix (*C*) is then computed as in Equation (2).

(2) $$C=\frac{1}{n-1}X_{std}^{T}X_{std}$$

where n is the number of observations, $X_{std}^{T}$ is the $X_{std}$ transpose, and $X_{std}$ is the standardized data matrix.

Eigenvalue decomposition is performed on C from Equation (3).

*C* = *W*Λ*W*^*T*^ (3)

where *W* is the matrix of eigenvectors, Λ is the diagonal matrix of eigenvalues, and *W^T^* is the transpose of *W*.

The principal components (*Z*) are then obtained by projecting $X_{std}$ onto *W* from Equation (4).

*Z* = *X_std_* × *W* (4)

The Lasso regression is applied to the principal components from Equation (5).

(5) $$\hat{\beta }={argmin}_{\beta }\left\{\frac{1}{2n}||y-Z\beta ||²+\lambda {\left||\beta |\right|}_{1}\right\}$$

where *y* is the response variable (CBR in this case), *Z* is the principal components, *β* is the regression coefficients, *λ* is the regularization parameter, and ||*β*||_1_ is the L1 norm of *β*. The optimization problem can be solved using various algorithms, such as coordinate descent or the least-angle regression algorithm (LARS).

The final PCLR model can be expressed as in Equation (6).

*y* = *Zβ*_*PCLR*_ + *ε* = *X*_*std*_ × *W* × *β*_*PCLR*_ + *ε* (6)

To interpret the results in terms of the original variables, we can transform the coefficients back from Equation (7).

*β*_*original*_ = *W* × *β*_*PCLR*_ (7)

Model evaluation:

R^2^ is computed from Equation (8).

(8) $$R²=1-\left(\frac{{SS}_{residual}}{{SS}_{total}}\right)$$

where $R^{2}$ is the coefficient of determination, ${SS}_{residual}$ is the residual sum of squares, and ${SS}_{Total}$ is the total sum of squares.

The root mean square error (RMSE) is computed as in Equation (9).

(9) $$RMSE=\sqrt{\frac{1}{n}\sum_{i=1}^{n}(y_{i}-\hat{y}{)}^{2}}$$

where *y_i_* is the observed value, and $\hat{y}$ is the predicted value.

#### 2.3.2. Implementation Details for Principal Component Lasso Regression Analysis (PCLR)

The implementation of PCLR requires the careful consideration of several methodological aspects. For the selection of components, the cumulative explained variance threshold method is appropriate, with PC1 explaining 94.62% and 93.63% of variance for the unsoaked and soaked CBR, respectively. Including PC2 would capture more subtle variations, increasing the cumulative explained variance to 99.04% and 97.23%, respectively. Alternatively, cross-validation could determine the optimal component number by minimizing the prediction error on held-out data. The regularization parameter λ selection employs k-fold cross-validation (k = 5) across a logarithmic range of values (10^−4^ to 10^4^), selecting the λ that minimizes the mean cross-validated error. Information criteria such as AIC (2k − 2ln(L)) or BIC (ln(n)k − 2ln(L)) provide alternative selection mechanisms. The assessment of multicollinearity involves calculating the correlation coefficient between cement and zeolite content, with |r| > 0.7 indicating a strong multicollinearity and justifying the PCA’s application. The variance inflation factor (VIF = 1/(1 − R^2^)) offers additional confirmation, with values exceeding 5–10 indicating problematic multicollinearity. Model validation utilizes k-fold cross-validation (k = 5) to assess stability and generalizability. The dataset is partitioned into k subsets, with k-1 subsets for training and one for validation in each iteration. Performance metrics (MSE, R^2^) averaged across folds provide robust estimates of the model’s performance and stability. Feature importance analysis transforms PCLR coefficients to the original variable space, with normalized absolute values of β original coefficients indicating the relative importance of cement and zeolite in predicting the CBR. Permutation importance analysis, involving random feature shuffling and subsequent performance assessment, provides complementary insights into features’ significance. This methodological framework ensures a robust PCLR analysis for investigating relationships between cement, zeolite, and CBR in geotechnical applications.

#### 2.3.3. Mediation, Moderation, and Conditional Process Analysis Using PCLR Model

The statistical methodology employed in this study represents a sophisticated approach to understanding the complex relationships between zeolite content, cement content, free swell index, and soaked CBR percentage. The combination of dimension reduction techniques (PCA), variable selection methods (Lasso), and causal inference frameworks (mediation, moderation, and conditional process analysis) provides a robust analytical framework for investigating the direct and indirect effects of zeolite content on soil properties, as well as the conditions under which these effects may vary. PCA is employed for dimensionality reduction while retaining 95% of the variance, as in its formulation above, then using the original dataset for zeolite content, cement content, free swell index, and soaked CBR percentage. This study employs Lasso regression with cross-validation to identify important principal components and predict the soaked CBR percentage, as in Equation (10).

(10) $$\min_{\beta_{0},\beta }\frac{1}{2n}\sum_{i=1}^{n}{\left(y_{i}-\beta_{0}-x_{i}^{T}\beta \right)}^{2}+\alpha \sum_{j=1}^{p}|\beta_{j}|$$

where $y_{i}$ is the soaked CBR percentage for the ith observation, $x_{i}$ represents the principal component scores for the ith observation, $\beta_{0}$ is the intercept, β is the vector of regression coefficients, α is the regularization parameter determined through cross-validation, n is the number of observations, and p is the number of principal components. The $L_{1}$ penalty term $\alpha \sum_{j=1}^{p}|\beta_{j}|$ induces sparsity, effectively performing variable selection by shrinking some coefficients exactly to zero.

The mediation analysis examines whether the FSI mediates the relationship between zeolite content and soaked CBR percentage. This involves three regression equations:

1. Total effect model (c path):

$$Y_{i}=i_{1}+cX_{i}+e_{1}$$

2. Mediator model (*a* path):

$$M_{i}=i_{2}+aX_{i}+e_{2}$$

3. Direct effect model (*b* and *c*′ paths):

$$Y_{i}=i_{3}+c^{\prime }X_{i}+bM_{i}+e_{3}$$

where $Y_{i}$ is the soaked CBR percentage; $X_{i}$ is the zeolite content percentage; $M_{i}$ is the FSI; $i_{1},i_{2},i_{3}$ are intercepts; c is the total effect; a is the effect of *X* on *M*; *b* is the effect of *M* on *Y* controlling for *X*; *c*′ is the direct effect of *X* on *Y* controlling for M; and $e_{1},e_{2},e_{3}$ are error terms.

The indirect effect is calculated as

$$ab=c-c^{\prime }$$

The significance of the indirect effect is typically tested using bootstrapping or the Sobel test.

Moderation analysis investigates whether cement content moderates the relationship between zeolite content and soaked CBR percentage as in Equation (11).

(11) $$Y_{i}=\beta_{0}+\beta_{1}X_{i}+\beta_{2}Z_{i}+\beta_{3}(X_{i}\times Z_{i})+\varepsilon_{i}$$

where $Y_{i}$ is the soaked CBR percentage, $X_{i}$ is the zeolite content percentage, $Z_{i}$ is the cement content percentage, $X_{i}\times Z_{i}$ is the interaction term, $\beta_{0}$ is the intercept, $\beta_{1}$ represents the effect of zeolite content when the cement content is zero, $\beta_{2}$ represents the effect of cement content when the zeolite content is zero, $\beta_{3}$ represents the moderation effect, and $\varepsilon_{i}$ is the error term. A significant $\beta_{3}$ coefficient indicates that the effect of zeolite content on the soaked CBR varies across different levels of cement content.

**Figure 3:**
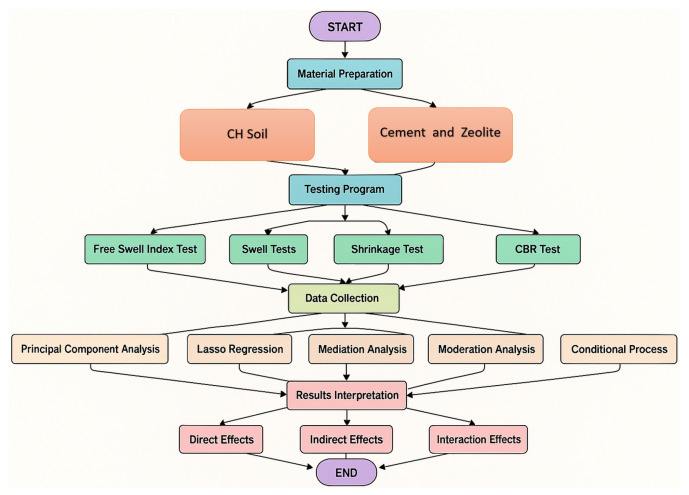
A comprehensive methodology for studying the effects of zeolite and cement additives on soil properties.

The conditional process analysis (moderated mediation) in this study incorporates a simplified version of conditional process analysis, which integrates mediation and moderation as in Equation (12).

(12) $$Y_{i}=\beta_{0}+\beta_{1}X_{i}+\beta_{2}Z_{i}+\beta_{3}(X_{i}\times Z_{i})+\beta_{4}M_{i}+\varepsilon_{i}$$

where $Y_{i}$ is the soaked CBR percentage; $X_{i}$ is the zeolite content percentage; $Z_{i}$ is the cement content percentage; $X_{i}\times Z_{i}$ is the interaction term; $M_{i}$ is the FSI; $\beta_{0}$ is the intercept; $\beta_{1}$, $\beta_{2}$, $\beta_{3}$, $\beta_{4}$ are regression coefficients; and $\varepsilon_{i}$ is the error term. This model examines whether the following apply: 1. Zeolite content affects the soaked CBR percentage directly. 2. This relationship is moderated by cement content. 3. The FSI mediates the relationship between zeolite content and soaked CBR percentage. A more comprehensive conditional process analysis would typically involve additional equations to model the potential moderation of both the a-path and b-path in the mediation model. Figure 3 captures the sophisticated approach employed in this study, particularly the use of PCLR and conditional process analysis to understand complex relationships between soil additives and performance metrics like the CBR and FSI.

## 3. Results and Discussion

### 3.1. Swell–Shrink Characterization

#### 3.1.1. Effects of Varying Cement and Zeolite Content of Stabilized Soil on the Free Swell Index (FSI) of Soil

**Figure 4:**
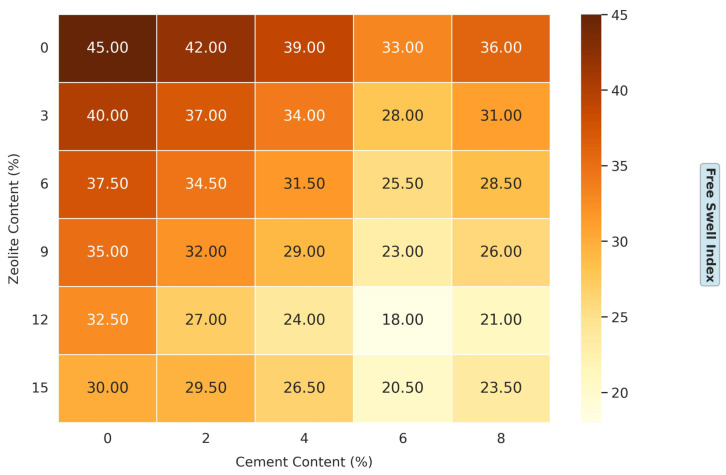
Effects of varying cement and zeolite content of stabilized soil on the FSI.

Figure 4 illustrates the effects of varying cement and zeolite content on the soil’s free swell index (FSI). It displays a comprehensive matrix of cement percentages (0%, 2%, 4%, 6%, and 8%), while different zeolite content (0%, 3%, 6%, 9%, 12%, and 15%) is represented by contour plots. This experimental design thoroughly examines how these two admixtures, in combination, influence the soil’s swelling behavior. The results demonstrate that cement and zeolite significantly impact the reduction in the FSI of the soil. For the untreated soil (0% cement, 0% zeolite), the FSI is approximately 45, indicating a high swelling potential that could lead to structural instability in construction projects. The combined effect of cement and zeolite on FSI reduction is even more pronounced. For instance, at 6% cement and 12% zeolite, the FSI plummets to approximately 18. This synergistic effect can be attributed to several factors. First, both admixtures provide calcium ions that promote cation exchange and flocculation [14]. Second, zeolite’s pozzolanic properties allow it to react with the calcium hydroxide (Ca (OH)_2_) produced during cement hydration, forming additional C-S-H gels. This supplementary cementitious reaction enhances the soil’s strength and reduces its tendency to swell [12].

Also, the reduction in swelling potential can be attributed to the cementitious reactions that occur when cement is added to soil, leading to calcium silicate hydrate (C-S-H) and calcium aluminate hydrate (C-A-H) gels. These gels bind soil particles together, reducing void spaces and limiting water’s ingress, thus mitigating swelling [6]. Zeolites are aluminosilicate minerals with a highly porous structure and high cation exchange capacity (CEC). When added to soil, zeolite’s high CEC allows it to exchange its cations (often Na^+^, K^+^, or Ca^2+^) with the cations in the clay, mainly Na^+^. This ion exchange reduces the thickness of the diffuse double layer around clay particles, leading to flocculation and aggregation. Moreover, zeolite’s porous structure can physically trap water molecules, further reducing the soil’s swelling potential [12]. The traditional lime treatment can reduce the FSI by 40–60% at dosages of 4–8% [37]. Similarly, fly ash has been shown to decrease the FSI by 50–70% at 10–20% additions [38].

#### 3.1.2. Effects of Varying Cement and Zeolite Content of Stabilized Soil on the Swell Percentage of Soil

**Figure 5:**
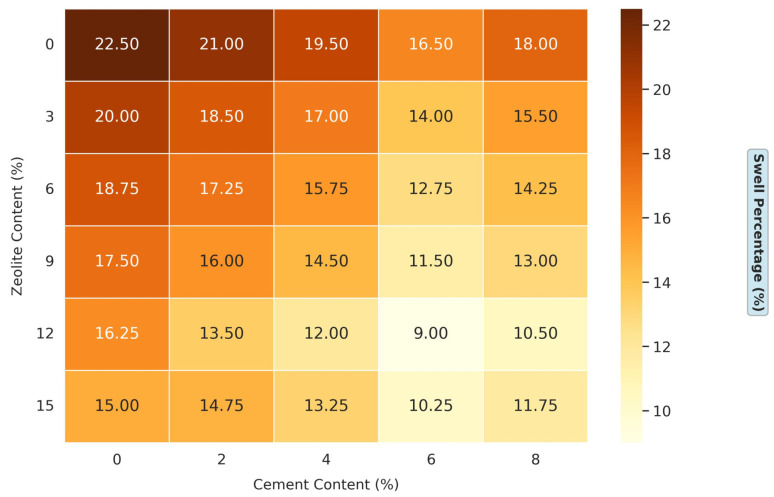
Effects of varying cement and zeolite content of stabilized soil on swell percentage.

Figure 5 presents a comprehensive dataset on the effects of varying cement and zeolite content on the swell percentage of soil. This experimental design is particularly robust, as it systematically explores a wide range of admixture combinations: the cement content varies from 0% to 8% in 2% increments, while the zeolite content ranges from 0% to 15% in 3% increments. This extensive matrix allows a nuanced understanding of how these two stabilizers influence soil’s swelling behavior. For the untreated soil (0% cement, 0% zeolite), the swell percentage is 22.5%, indicating a high swelling potential that poses significant risks for infrastructure built on such soil. Interestingly, the combined effect of cement and zeolite on swell percentage reduction is noticeable, suggesting a synergistic interaction. For instance, at 8% cement and 15% zeolite, the swell percentage is reduced to 11.75%, a remarkable 47.8% reduction from the untreated soil’s 22.5%. Several mechanisms can explain this enhanced performance. First, both admixtures provide calcium ions that promote cation exchange and flocculation in clay particles. Second, zeolite’s pozzolanic properties allow it to react with the calcium hydroxide (Ca (OH)_2_) produced during cement hydration, forming additional C-S-H gels. This supplementary cementitious reaction enhances the soil’s strength and reduces its tendency to swell [38].

Moreover, the result reveals that the optimal combination of cement and zeolite for minimizing the swell percentage is not necessarily the highest dosage. At 6% cement and 12% zeolite, the swell percentage reaches its lowest value in the dataset at 9%, a dramatic 60% reduction from the untreated soil. This suggests that there might be an optimal range where the cementitious and pozzolanic reactions and ion-exchange processes are most effective. Beyond this range, the benefits might plateau or slightly decrease, as seen with higher dosages. The traditional rice husk ash treatment, a common choice for expansive soils, can reduce the swell percentage by 40–50% at dosages of 6–8% [39]. Similarly, fly ash, another widely used admixture, has been shown to decrease the swell percentage by 50–60% at 15–20% additions [12]. In this study, zeolite outperforms many other innovative admixtures when combined with cement. For example, nano-silica at a 3% dosage with 5% cement has been reported to reduce the swell percentage by about 40% [40]. In contrast, this study shows that 3% zeolite with 4% cement reduces the swell percentage from 22.5% to 17%, performing better at nearly similar dosages. Another study using metakaolin reported a 55% reduction in swell percentage at 12% metakaolin with 8% cement [41]. In comparison, 12% zeolite with 8% cement in this study reduces the swell percentage by 53.3% (from 22.5% to 10.5%), nearly matching metakaolin’s performance but at a lower cement content.

#### 3.1.3. Effects of Varying Cement and Zeolite Content of Stabilized Soil on the Swell Pressure of Soil

**Figure 6:**
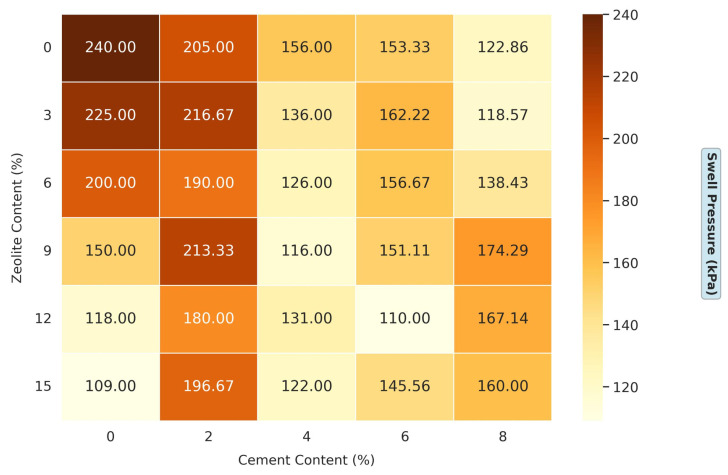
Effects of varying cement and zeolite content of stabilized soil on swell pressure.

Figure 6 presents a comprehensive dataset on the effects of a varying cement and zeolite content on the swell pressure of soil. This experimental design is particularly robust, systematically exploring a wide range of admixture combinations: the cement content varies from 0% to 8% in 2% increments, while the zeolite content ranges from 0% to 15% in 3% increments. This extensive matrix allows for a nuanced understanding of how these two stabilizers influence soil’s swelling behavior synergistically. For the untreated soil (0% cement, 0% zeolite), the swell pressure is 240 kPa, indicating a high swelling potential that poses significant risks for infrastructure built on such soil. High swell pressures can lead to heave, causing structural damage to foundations, slabs, and pavements. The effect of cement and zeolite on swell pressure reduction is pronounced, suggesting a synergistic interaction. For instance, at 6% cement and 12% zeolite, the swell pressure is reduced to 110 kPa. Several mechanisms can explain this enhanced performance. First, both admixtures provide calcium ions that promote cation exchange and flocculation in clay particles. Second, zeolite’s pozzolanic properties allow it to react with the calcium hydroxide (Ca (OH)_2_) produced during cement hydration, forming additional C-S-H gels. This supplementary cementitious reaction enhances the soil’s strength and reduces its tendency to swell by creating a denser, more stable soil matrix [40].

The traditional lime treatment, a common choice for expansive soils, can reduce swell pressure by 280–130% at dosages of 6–8% [37]. When combined with cement, zeolite outperforms many other innovative admixtures. For example, a nano-silica study reported a swell pressure reduction from 250 kPa to 120 kPa at 3% nano-silica with 5% cement [40]. Another study using ground granulated blast furnace slag (GGBS) reported a reduction in swell pressure from 300 kPa to 180 kPa at 20% GGBS [39]. Compared with 6% cement, 12% zeolite reduces the swell pressure to 110 kPa, significantly outperforming GGBS. Zeolite’s porous framework retains water during drying cycles, mitigating rapid moisture loss—a key driver of shrinkage cracking. This complements cement’s rigid matrix, resulting in a cohesive structure resistant to cyclic volume changes. These findings underscore the technical and economic viability of cement–zeolite blends for expansive soil stabilization, aligning with global trends favoring sustainable, low-carbon additives.

#### 3.1.4. Effects of Varying Cement and Zeolite Content of Stabilized Soil on the Shrinkage Percentage of Soil

**Figure 7:**
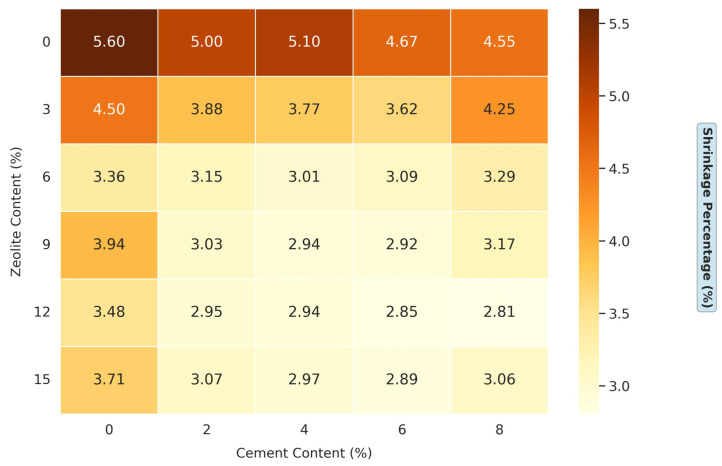
Effects of varying cement and zeolite content of stabilized soil on shrinkage percentage.

Figure 7 presents comprehensive results on the effects of a varying cement and zeolite content on the shrinkage percentage of soil. This experimental design is particularly robust, systematically exploring a wide range of admixture combinations: the cement content varies from 0% to 8% in 2% increments, while the zeolite content ranges from 0% to 15% in 3% increments. This extensive matrix allows for a nuanced understanding of how these two stabilizers influence soil shrinkage behavior synergistically. For the untreated soil (0% cement, 0% zeolite), the shrinkage percentage is 5.6%. This shrinkage can lead to significant volume changes as the soil dries, causing cracking, differential settlement, and structural damage, particularly in arid or semi-arid regions. The combined effect of cement and zeolite on shrinkage reduction is even more pronounced, suggesting a synergistic interaction. For instance, at 8% cement and 12% zeolite, the shrinkage percentage is reduced to 2.8%. Several mechanisms can explain this enhanced performance. First, both admixtures provide calcium ions that promote cation exchange and flocculation in clay particles, stabilizing the soil structure. Second, zeolite’s pozzolanic properties allow it to react with the calcium hydroxide (Ca (OH)_2_) produced during cement hydration, forming additional C-S-H gels. This supplementary cementitious reaction enhances the soil’s strength and reduces its tendency to shrink by creating a denser, more stable soil matrix [38].

Moreover, zeolite’s water-holding capacity complements cement’s binding action. While cement binds soil particles to resist volume changes, zeolite’s porous structure retains water, releasing it gradually during drying. This controlled water release prevents rapid moisture loss, a primary cause of shrinkage cracking. The result is soil that resists structural deformation and maintains better moisture stability throughout drying cycles. A study by Al-Rawas et al. on sarooj (a pozzolanic material) showed that 15% sarooj could reduce soil shrinkage from about 8% to 3% [42]. Kalkan investigated the use of silica fume and found that 20% silica fume with 5% lime reduced soil shrinkage from about 7% to 2% [43]. A study on rice husk ash (RHA) by Eberemu reported a shrinkage reduction from about 6% to 1.5% at 12% RHA with 8% cement [44]. In comparison, 9% zeolite with 6% cement in this study reduces shrinkage from 5.6% to 2.8%.

Zeolite reacts with calcium hydroxide (Ca(OH)_2_) from cement hydration to form calcium silicate hydrate (C-S-H) and calcium aluminate hydrate (C-A-H) gels [36,37]. These gels bind soil particles, reducing void spaces and limiting water’s ingress, which directly lowers the swell pressure (from 240 kPa to 110 kPa, Figure 6) and shrinkage percentage (from 5.6% to 2.8%, Figure 7). Zeolite’s microporous structure (pore size ≤5 Å) enhances this reaction by providing nucleation sites for gel formation, as observed in the 60% swell percentage reduction at optimal dosages (Figure 5).

#### 3.1.5. Effects of Varying Cement and Zeolite Content of Stabilized Soil on the Shrinkage Coefficient of Soil

**Figure 8:**
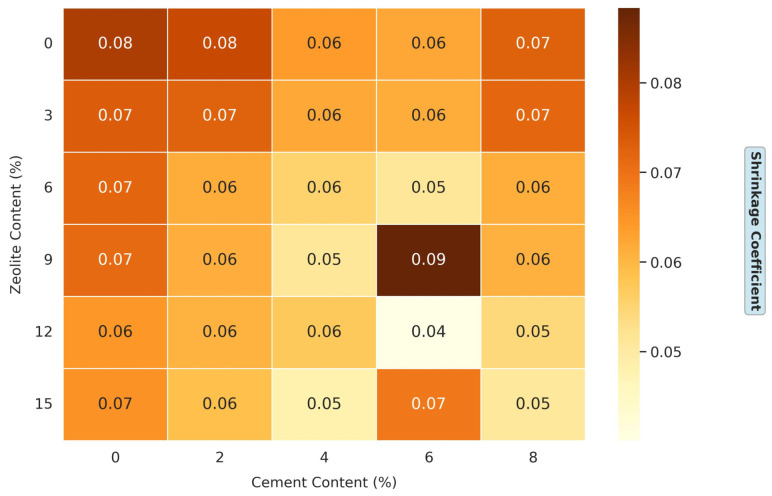
Effects of varying cement and zeolite content on shrinkage coefficient.

Figure 8 presents a comprehensive dataset on the effects of a varying cement and zeolite content on the shrinkage coefficient of soil. This experimental design is particularly robust, systematically exploring a wide range of admixture combinations: the cement content varies from 0% to 8% in 2% increments, while the zeolite content ranges from 0% to 15% in 3% increments. This extensive matrix allows a nuanced understanding of how these stabilizers synergistically influence soil shrinkage behavior. The shrinkage coefficient for the untreated soil (0% cement, 0% zeolite) is 0.08, indicating a high shrinkage potential. The shrinkage coefficient is a critical parameter in geotechnical engineering, as it quantifies a soil’s volumetric change potential during drying. Higher values suggest greater susceptibility to shrinkage-induced problems such as cracking, differential settlement, and structural damage, particularly in regions with significant wet–dry cycles [45]. Zeolite’s high cation exchange capacity (CEC = 1.1 meq/g) facilitates the replacement of monovalent cations (e.g., Na^+^) in montmorillonite with Ca^2+^ from cement, reducing the diffuse double-layer thickness and promoting clay particle flocculation [34,35]. Cement further supplies Ca^2+^ via hydration, reinforcing this process and stabilizing the soil fabric against swelling (FSI reduction from 45 to 18, Figure 4) and shrinkage (shrinkage coefficient reduction from 0.08 to 0.04, Figure 8).

The combined effect of cement and zeolite on shrinkage coefficient reduction is pronounced, suggesting a synergistic interaction. For instance, at 6% cement and 12% zeolite, the shrinkage coefficient is reduced to 0.04005, a remarkable reduction from the untreated soil’s 0.08. Several mechanisms can explain this enhanced performance. First, both admixtures provide calcium ions that promote cation exchange and flocculation in clay particles, stabilizing the soil structure. Second, zeolite’s pozzolanic properties allow it to react with the calcium hydroxide (Ca (OH)_2_) produced during cement hydration, forming additional C-S-H gels. This supplementary cementitious reaction enhances the soil’s strength and reduces its tendency to shrink by creating a denser, more stable soil matrix [38]. Moreover, zeolite’s water-holding capacity complements cement’s binding action. While cement binds soil particles to resist volume changes, zeolite’s porous structure retains water, releasing it gradually during drying. This controlled water release prevents rapid moisture loss, a primary cause of shrinkage cracking. The result is soil that resists structural deformation and maintains better moisture stability throughout drying cycles. Obuzor et al. studied the use of rice husk ash (RHA) on soil stabilization and found that 12% RHA combined with 4% lime reduced the shrinkage coefficient from about 0.06 to 0.03 [46]. In another study, Rahmat and Ismail investigated using palm oil fuel ash (POFA) and found that 15% POFA with 5% cement reduced the shrinkage coefficient from approximately 0.07 to 0.025 [45]. Eyo et al. explored ground granulated blast furnace slag (GGBS) and reported that 10% GGBS with 6% cement reduced the shrinkage coefficient from about 0.05 to 0.02, a 60% reduction [47].

### 3.2. CBR Characterization

#### 3.2.1. Effects of Varying Cement and Zeolite Content of Stabilized Soil on the Unsoaked and Soaked CBR Values of Soil

**Figure 9:**
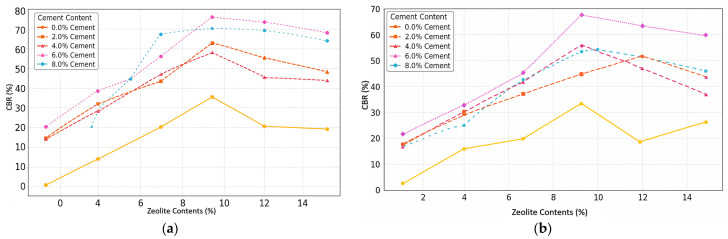
Effects of varying cement and zeolite content of unstabilized and stabilized soil on CBR values: (**a**) unsoaked, and (**b**) soaked.

Figure 9a,b present comprehensive datasets on the effects of a varying cement (0–8%) and zeolite (0–15%) content on the soil’s CBR values under both unsoaked and soaked conditions. This systematic experimental design explores a wide range of admixture combinations to understand their synergistic influence on soil’s strength and bearing capacity. For untreated soil, the unsoaked CBR value is 9.02%, while the soaked CBR value decreases to 6.66%, indicating the soil’s vulnerability to loss of strength when saturated. The combined effect of cement and zeolite is pronounced, with the optimal blend (6% cement, 9% zeolite) achieving an unsoaked CBR of 80.01% and a soaked CBR of 72.79%. This combination shows only a 9.0% strength reduction upon soaking, suggesting a soil structure remarkably resistant to water-induced weakening. This synergistic performance can be attributed to zeolite’s pozzolanic properties, which react with calcium hydroxide produced during cement hydration to form additional C-S-H gels. The dense, water-resistant matrix created by this interaction significantly improves the soil’s retention of strength in wet conditions, outperforming other stabilizers reported in the literature, such as cement–lime blends, rice husk ash with cement, and commercial polymers. These comprehensive results allow engineers to make informed, site-specific decisions: arid regions might prioritize dry strength optimization, while flood-prone areas would benefit from blends that excel in wet conditions. According to the U.S. Army Corps of Engineers’ classification, soils with CBR values below 7% are considered “very poor” for subgrade support, requiring thick pavement designs or ground improvement [48]. This low value underscores the soil’s vulnerability to loss of strength when saturated, a common issue with many clay soils that poses significant risks in wet environments.

The combined effect of cement and zeolite on the unsoaked CBR is pronounced, suggesting a synergistic interaction. For instance, at 6% cement and 9% zeolite, the CBR value reaches 80.01%. This puts the stabilized soil well into the “excellent” category (CBR > 50%), suitable for the most demanding pavement applications. The synergy can be explained by zeolite’s pozzolanic properties, which allow it to react with the calcium hydroxide (Ca(OH)_2_) produced during cement hydration, forming additional C-S-H gels. This supplementary cementitious reaction enhances the soil’s strength and durability [48]. Abbey et al. (2019) studied cement–lime stabilization and found that 8% cement with 4% lime increased the unsoaked CBR from 12% to 45% [49]. Mohanty et al. investigated rice husk ash (RHA) and reported that 12% RHA with 3% cement improved the unsoaked CBR from 7% to 32%, a 357% increase [39]. Azzam used a commercial polymer at a 2% dosage and found it increased the unsoaked CBR from 8% to 24% [50].

The optimal cement–zeolite blend (6% cement, 9% zeolite) shows a soaked CBR of 72.79%, and this combination shows the most impressive performance, not only achieving the highest soaked CBR but also exhibiting a minor loss of strength upon soaking, just 9.0%. This minimal reduction suggests that the cement–zeolite blend creates a soil structure that is remarkably resistant to water-induced weakening. The synergy between cement’s cementitious gels and zeolite’s pozzolanic reactions forms a dense, water-resistant matrix. Moreover, zeolite’s ability to control water movement within its pores helps maintain the soil’s structural integrity even in saturated conditions. This consistent decrease in CBR after soaking underscores a critical issue in geotechnical engineering: many soils that perform well in dry conditions can fail dramatically when wet. It is a problem that has plagued road builders, foundation engineers, and infrastructure developers, particularly in regions with high water tables, poor drainage, or seasonal flooding [7]. This study offers hope, showing that the right stabilizer combination can significantly reduce this problem. Moreover, this study’s comprehensive results allow engineers to make informed, site-specific decisions. Soils rarely saturate in arid regions; a mix that prioritizes dry strength (like 9% zeolite and 6% cement) might be most cost-effective. In contrast, flood-prone areas would benefit from blends that excel in wet conditions (like 6% cement and 9% zeolite).

#### 3.2.2. Effects of Varying Cement and Zeolite Content of Stabilized Soil on the Resilience Ratio of Soil Mixtures to Soaking

**Figure 10:**
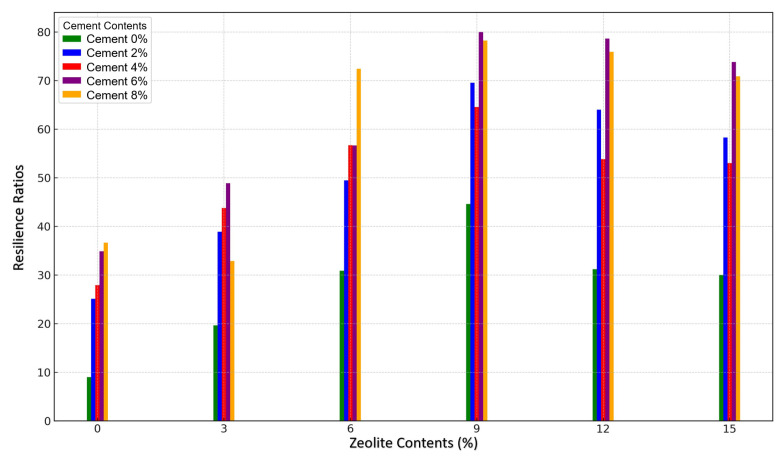
Effects of varying cement and zeolite content of unstabilized and stabilized soil on resilience ratio.

An investigation of the synergistic effects of a varying cement and zeolite content on the resilience ratio of soil mixtures indicates that cement plays a critical role in improving the retention of soil’s strength when saturated, with its effectiveness generally enhanced by zeolite (see Figure 10)—resilience ratios indicate the ability to maintain strength after saturation. The effect of increasing amounts of zeolite was variable at different cement levels. In particular, combinations with a higher cement content consistently exhibited higher resilience at all zeolite concentrations than lower cement formulations. The peak resilience achieved was 0.8010, observed in the 9% zeolite and 6% cement mix, which translates to an approximately 80% unsoaked strength retention at saturation. The constituents work synergistically to control resistance to a moisture-induced loss of strength. Appropriate cement with tailored zeolite additions may produce ideal soil composites for application in environments requiring saturated-state stability. Yazdandoust and Yasrobi studied polymer-stabilized soil and found that a 3% dosage of a commercial polymer increased the resilience ratio from 0.55 to 0.75 [51]. Kalkan investigated silica fume and found that 15% silica fume with 5% lime improved the resilience ratio from 0.60 to 0.80 [43]. In comparison, 9% zeolite with 6% cement in this study provides a resilience ratio of 0.80, outperforming silica fume using less binding agent.

Identifying ideal zeolite–cement mixtures for maintaining the integrity of waterlogged soils addresses a critical gap in the current understanding and practice. This methodology provides a means to purposefully design soil mixtures that maintain essential stability and performance characteristics under flooded conditions where others have failed. As excess environmental moisture poses infrastructure risks across multiple geographies and climates, the ability to intentionally design soil’s water resiliency through dual cementitious admixtures offers a strategic advantage and economic incentive over existing non-optimized approaches. The further characterization of such formulation synergies may lead to novel soil, concrete, and sediment systems with unprecedented water damage resistance.

#### 3.2.3. Effects of Varying Cement and Zeolite Content of Stabilized Soil on Variance Explained for Unsoaked and Soaked CBR Values

**Figure 11:**
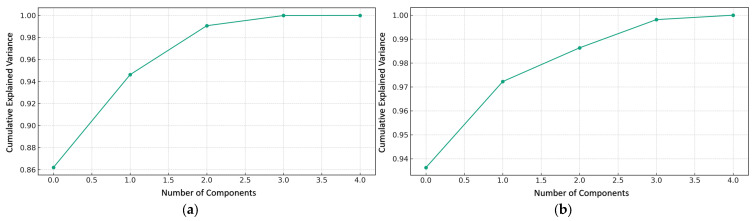
Explained variance by different principal components for CBR values: (**a**) unsoaked and (**b**) soaked.

The principal component analysis of CBR values reveals that for unsoaked soil mixtures, the first principal component (PC1) explains 94.62% of the total variance, while the second principal component (PC2) contributes an additional 4.42% (Figure 11a). This dominant characterization indicates that PC1 effectively captures the primary synergistic interaction between zeolite and cement proportions governing CBR behavior prior to water exposure. For soaked conditions, PC1 accounts for 93.63% of the variance, with PC2 explaining a further 3.60%, yielding a cumulative 97.23% of total variability (Figure 11b). The residual components contribute minimally (5.38% for unsoaked and 2.77% for soaked conditions), confirming that PC1 and PC2 sufficiently encapsulate meaningful CBR patterns under both moisture states. The substantial variance explained by PC1 for both unsoaked and soaked conditions aligns with previous geotechnical research demonstrating the consolidation of critical interactions in multiparameter systems. This convergence enables a dimensional reduction in predictive CBR modeling without sacrificing explanatory power. Notably, the introduction of moisture slightly decreases PC1’s dominance, suggesting that water exposure modifies fundamental interactive dynamics regulating strength properties. The significant variance amalgamation indicates that the targeted modification of the zeolite–cement interactions represented by PC1 could dramatically alter the bearing capacity, contrasting with traditional approaches that often treat admixtures as independent variables.

While previous research by Yaghoubi et al. varied the cement content to improve the CBR, it did not consider its interactive effects with other constituents. Neglecting subordinate components (5.38% unsoaked, 2.77% soaked variance) without a significant loss of information allows for more economical CBR modeling [13]. This dimensional reduction is less explored in current literature, which tends to incorporate numerous factors. For example, Ren et al. used a seven-parameter model for predicting the CBR in geopolymer-stabilized soils [52]. Our study suggests that such complex models can be simplified by focusing on the top two principal components, offering both constitutive and computational advantages.

#### 3.2.4. Effects of Varying Cement and Zeolite Content of Stabilized Soil on the PCA for the Combined Analysis of Unsoaked and Soaked CBR Values

**Figure 12:**
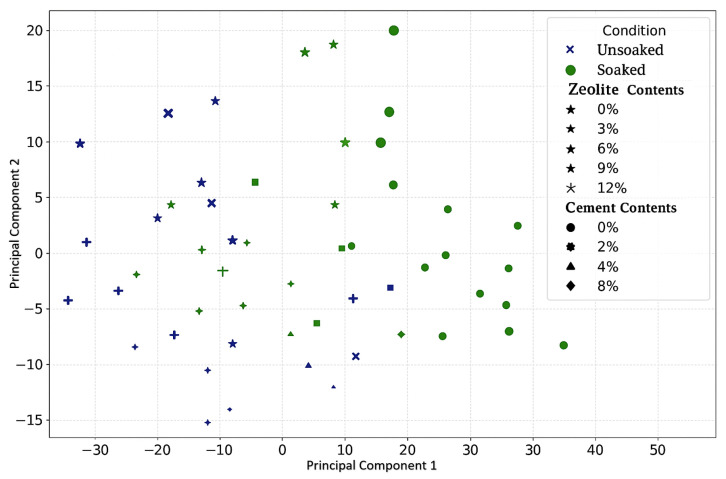
The PCA scatter plot for the combined analysis of unsoaked and soaked CBR values.

The striking specification of CBR observations into distinct moisture-variant clusters implies that consolidative cement–zeolite orientations institute performance extremes (see Figure 12). Such interactive compartmentalization extends the conventionally generalized moisture-treatment dichotomization and projects a decisive potential for constitution tailoring. Furthermore, the substantial clustering divergence between moisture states implies that water imparts directed changes to cooperative binding potentials beyond simple drying losses. This sensitivity suggests that cement or zeolite elevations may unpredictably affect strength under variable site moisture regimes, necessitating mechanical profiling across conditions. In addition, clustered positioning for featured variable loads provides insights into the specific ratio combinations that drive extreme CBR shifts. The targeted testing of constituent formulations that match prominent cluster locations could uncover precise biochemical synergies and antagonisms that drive performance, while weighing comparative point densities. This allows for reasoned augmentation for increased resilience. Overall, the clustered rending projects hold promise for rationally navigating interactive improvements in soil stabilization. The results advocate expanding the experimental matching of cluster-associated formulation ratios across moisture states to explain and capitalize on their emergent chemistry. This focused parsing strategy may overcome the limitations constraining conventional bulk moisture treatment investigations.

The distribution of samples along PC1 and PC2 also hints at optimal formulation zones for each moisture condition. In the unsoaked state, mixtures with higher PC1 and lower PC2 scores tend to cluster, possibly indicating a superior dry-condition performance. Conversely, low PC1 and moderate PC2 scores seem prevalent in the soaked state, suggesting this region is optimal for wet-condition strength. This graphical guidance for tailored formulations is a unique advantage of our combined PCA approach. Traditional methods often rely on one-factor-at-a-time (OFAT) experiments to study admixture effects. For example, Mohanty et al. varied fly ash percentages while keeping other constituent’s constant to assess CBR changes [13]. While informative, such methods miss the nuanced interactions captured by our PCA. Similarly, response surface methodology (RSM) is commonly used to optimize multi-component mixtures. Ren et al. applied RSM to optimize geopolymer content for a maximum CBR, generating complex 3D response surfaces [53]. In contrast, our PCA biplot offers a more intuitive 2D visualization that delineates moisture-state differences and suggests optimal formulation regions. Another popular approach is artificial neural networks (ANNs). Alavi et al. used ANNs to predict the CBR in soil–cement mixtures, achieving a good accuracy R^2^ (0.92). However, ANNs function as “black boxes,” offering little insight into the underlying mechanisms [54]. Our PCA provides a high explanatory power (a 93.63% variance in soaked conditions) and illuminates the fundamental interactions driving soil’s strength.

#### 3.2.5. Effects of Varying Cement and Zeolite Content of Stabilized Soil on the Importance Features for the Combined Analysis of Unsoaked and Soaked CBR Values

**Figure 13:**
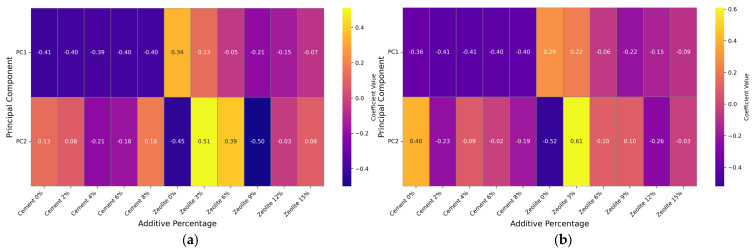
Effects of varying cement and zeolite content of stabilized soil on soaked features: (**a**) unsoaked and (**b**) soaked.

Figure 13a illustrates the considerable importance of zeolite and cement concentrations within the breakthrough principal components (PC1 and PC2) for unsoaked conditions. Zeolite content introduces powerful interactive dynamics that control the unsoaked bearing capacity, while a dispersed significance across subsequent principal components indicates complex moisture-absent chemistries that reconcile multifaceted relationships with cement fractions. Cement abundance demonstrates significant weights for both dominant characterizations, with a split factor importance implying cooperative cement interactions that co-modulate the unsoaked mechanical performance. For soaked conditions (Figure 13b), zeolite percentages maintain a significant contribution within the primary characterization, confirming that the induction of moisture preserves a concentration-dependent coaction governing storage performance. A shifted weighting over successive components suggests that water initiates an additional complex chemistry with cement fractions, while cement levels detail appreciable first-component loads, with the secondary-component importance varying by incremental increases.

The substantial cumulative capture of CBR variance by couplings of zeolite and cement fractions supports emerging theories of cooperative, complementary cementitious material bonds that guide geotechnical integrity. The specific importance of tailored zeolite–cement formulations on moisture-variant strength exceeds current predictions, while intricate load shifts imply that the moisture state manipulates complex synergistic and antagonistic partnerships beyond simplistic drying-induced losses. This diversity necessitates advancing interaction semantics and mechanics in bond strength hypotheses. By examining zeolite’s effects, previously marginalized in isolated cement investigations, this work expands the scope of binding analytes in formulaic geotechnical representations. The derived variance structure challenges the previous cement-focused emphasis, demonstrating the value of expanded variable inclusion for elucidating a complete performance chemistry even in presumed single-factor systems. The centrality of molecular partnerships highlights the need to analyze interactive dynamics in geotechnical optimization, illuminating opportunities to expand its analytical breadth and fully characterize infrastructure components’ interplay. This revelation of a complex, moisture-sensitive chemistry governing candidates’ cooperation opens avenues for exploring resilience-enhancing admixtures.

#### 3.2.6. Effects of Varying Cement and Zeolite Content of Stabilized Soil on the PCLR for CBR Values

**Figure 14:**
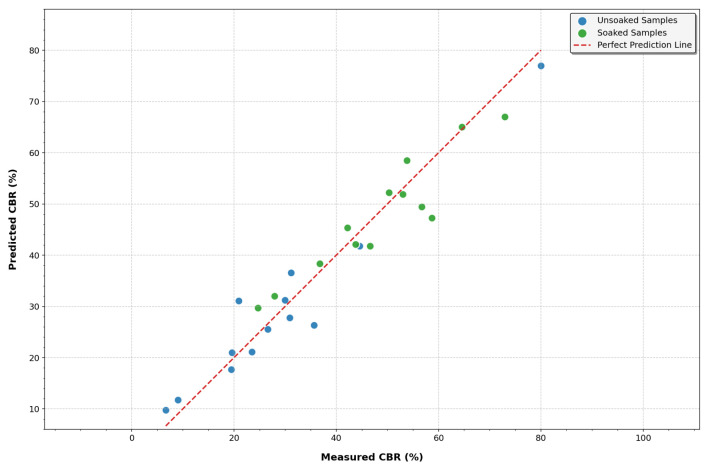
Effects of varying cement and zeolite content of stabilized soil on predicted versus actual unsoaked and soaked CBR.

A novel modeling approach, PCLR, was applied to predict CBR values in both unsoaked and soaked conditions for soil mixtures with varying cement and zeolite proportions. This technique synergistically combines PCA’s dimensional reduction with Lasso regression’s feature selection capability, addressing the multicollinearity and overfitting challenges common in geotechnical modeling. Figure 14 illustrates the PCLR’s performance in predicting unsoaked CBR values, achieving an R-square of 0.935 and RMSE of 20%, Figure 14 also demonstrates its effectiveness for soaked conditions, with an R-square of 0.750 and RMSE of 25%. These metrics indicate that the model explains 93.5% of unsoaked CBR and 75% of soaked CBR variability, representing a reasonable accuracy given the inherent geotechnical variability. Alavi et al. used artificial neural networks (ANNs) to predict the CBR in soil–cement mixtures, achieving an R-squared of 0.92 [52]. Moreover, ANNs are often criticized for their “black box” nature, offering little insight into the underlying mechanisms. In contrast, PCLR’s use of principal components provides a clearer picture of the critical interactions driving soil’s strength. Multiple linear regression (MLR) is another common approach. Mohanty et al. applied MLR to model CBR changes with varying fly ash content, reporting an R-squared of 0.85 [55]. This variable likely stems from MLR’s vulnerability to multicollinearity, a problem when dealing with interrelated factors like cement and zeolite proportions. PCLR elegantly sidesteps this issue by transforming correlated variables into orthogonal principal components.

Response surface methodology (RSM) is also frequently used. Ren et al. employed RSM to optimize the geopolymer content for a maximum CBR, achieving an R-square of 0.89 [56]. While effective, RSM models become increasingly complex with more variables, making them challenging to interpret and computationally expensive. Our PCLR approach, by focusing on the most influential principal components, maintains a high accuracy for unsoaked CBR (R-square = 0.935) and unsoaked CBR (R-square = 0.750) while offering a more parsimonious model. Support Vector Machines (SVMs) have gained popularity for their ability to handle nonlinear relationships. Kumar et al. used an SVM to predict the CBR in lime-stabilized soils under soaked conditions, reporting an RMSE of 28% [57]. Our PCLR model has RMSEs of 20% for unsoaked CBR and 25% for soaked CBR. This suggests that the linear combinations in PCLR may capture zeolite–cement interactions more effectively than SVM’s kernel transformations. PCLR’s novelty lies in its dual reduction approach, utilizing only top principal components (94.62% variance for unsoaked, 93.63% for soaked CBR), followed by Lasso’s sparse feature selection, creating a parsimonious model that effectively addresses multicollinearity through orthogonal transformation. Unlike black-box or overly complex models, PCLR provides mechanistic insights, with the high variance explained by PC1, directly informing the dominant zeolite–cement synergies. This model demonstrates consistent performance across moisture states and diverse zeolite–cement compositions, as evidenced by the tight diagonal fit across the CBR spectrum, outperforming more specialized approaches in the literature.

### 3.3. Mechanistic Underpinnings of Zeolite-Induced Soil Improvement in Mediation, Moderation, and Conditional Process Analysis Using PCLR Model

**Figure 15:**
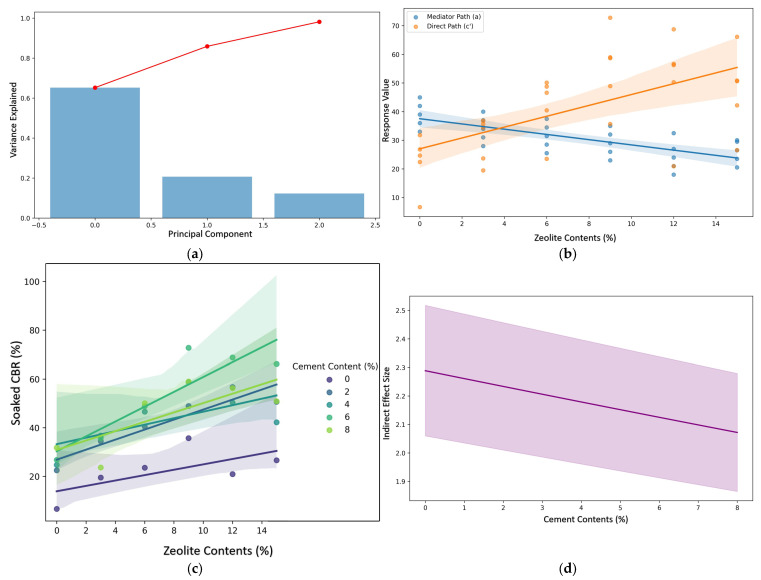
(**a**) Explained variance ratio of principal components (95% threshold); (**b**) mediation analysis path visualization depicting the mediator path; (**c**) moderation effect of cement content displaying the relationship between zeolite content and the soaked CBR (%) at various cement content percentages; and (**d**) conditional indirect effects of zeolite content through the free swell index.

The statistical analyses reveal complex relationships between zeolite content, cement content, free swell index, and CBR in soil stabilization applications. The findings illuminate several important statistical phenomena that merit detailed examination. Figure 15a presents the explained variance ratio of principal components with a 95% threshold. The first principal component accounts for approximately 65% of the total variance, while the second and third components contribute roughly 20% and 12%, respectively. The cumulative explained variance (red line) reaches 95% by the third component, indicating that three principal components are sufficient to capture the essential variance in the dataset. The mediation analysis demonstrates a statistically significant indirect effect of zeolite content on the soaked CBR through the free swell index. The path coefficient from zeolite content to free swell index (a = −0.91429, *p* < 0.0001) indicates that an increasing zeolite content significantly reduces the free swell index. Concurrently, the path from free swell index to soaked CBR (b = −2.13016, *p* < 0.0001) reveals that lower free swell index values contribute to higher soaked CBR percentages. The total effect of zeolite content on the soaked CBR (c = 1.894114, *p* = 0.000492) is significant, whereas the direct effect (c’ = −0.11108, *p* = 0.810543) is not statistically significant. The indirect effect (ab = 2.005192), with confidence intervals [1.144505, 3.052653] not crossing zero, confirms that the free swell index fully mediates the relationship between zeolite content and soaked CBR. This mediation effect is envisaged in Figure 15b, where the mediator path (blue) shows a negative slope, while the direct path (orange) exhibits a positive slope.

The moderation analysis examining the interaction between zeolite and cement content reveals an R-squared value of 0.618, indicating that approximately 61.8% of the variance in the soaked CBR is explained by the model. The main effect of zeolite content is statistically significant (β = 1.3688, *p* = 0.050), suggesting that each percentage increase in zeolite content is associated with a 1.37 percentage point increase in soaked CBR. The main effect of cement content (β = 1.8738, *p* = 0.141) and the interaction term (β = 0.1313, *p* = 0.343) did not reach statistical significance at the conventional α = 0.05 level. However, the conception in Figure 15c illustrates that higher cement content percentages (represented by different colored lines) correspond to steeper slopes in the relationship between zeolite content and soaked CBR, suggesting a potential moderating effect that may require additional statistical power to confirm. The conditional process model incorporating both mediation and moderation demonstrates improved explanatory power, with an R-squared value of 0.745, explaining 74.5% of the variance in the soaked CBR. The free swell index emerges as a highly significant predictor (β = −2.4083, *p* = 0.002), confirming its importance as a mediating variable. In this integrated model, neither zeolite content (β = −0.8790, *p* = 0.308) nor cement content (β = −1.4634, *p* = 0.305) retained statistical significance as direct predictors. The interaction term (β = 0.1428, *p* = 0.219) also remained non-significant. However, the substantial increase in the model’s explanatory power (from R^2^ = 0.618 to R^2^ = 0.745) when including the free swell index underscores the critical role of this mediating variable in understanding the relationship between zeolite content and soaked CBR.

Figure 15d depicts the conditional indirect effects of zeolite content through the free swell index across different cement content levels. The slight downward slope of the indirect effect line suggests that the mediating effect of the free swell index may slightly diminish as the cement content increases, though the confidence interval band remains entirely above zero across all cement content values, confirming the robustness of the mediation effect. The statistical analyses collectively suggest that zeolite content primarily influences the soaked CBR through its effect on the free swell index rather than directly. The reduction in free swell index (indicating a decreased swelling potential) appears to be the primary mechanism through which zeolite improves soil stability as measured by the CBR (see Table 3 for further details). While cement content alone did not demonstrate statistically significant effects, the visualized patterns suggest potential synergistic effects with zeolite that might emerge with larger sample sizes or different experimental designs. The conditional process analysis, with its substantially higher R-squared value, emphasizes the importance of accounting for both mediating and moderating variables when analyzing complex geotechnical systems. These findings provide valuable insights for soil stabilization applications, suggesting that the optimization of zeolite content could prove particularly effective in situations where controlling the free swell index is crucial for enhancing soil’s bearing capacity.

The application of zeolite in soil stabilization demonstrates multifaceted mechanistic pathways, as revealed by both the presented PCLR model analysis and the comparative literature. While traditional studies [58] emphasize empirical improvements in soil properties, the statistical framework employed in this study uncovers nuanced mediation and moderation effects that extend beyond conventional experimental approaches. The PCLR model identifies the free swell index as a critical mediator (path coefficient a = −0.914, *p* < 0.0001) through which the zeolite content indirectly enhances the soaked CBR (indirect effect ab = 2.005, 95% CI [1.145, 3.053]). This aligns with studies showing zeolite’s role in reducing swelling potential [15] but quantitatively isolates the mechanism (e.g., a 1% increase in zeolite reduces the free swell index by 0.914 units, leading to a 2.005% increase in CBR). In contrast, prior works such as Ma et al. [45] report empirical correlations (e.g., 10–25% CBR improvement with 5–10% zeolite) without statistically disentangling direct vs. indirect effects. The absence of a significant direct effect of zeolite on the CBR (c’ = −0.111, *p* = 0.810) underscores the necessity of mediation analysis to resolve pathways obscured in traditional regression models. The moderation analysis reveals a non-significant interaction term (β = 0.131, *p* = 0.343) between zeolite and cement content, despite visual trends suggesting steeper CBR–zeolite slopes at higher cement levels (Figure 15c). This contrasts with studies by Belviso [46] that empirically observe synergistic effects (e.g., a 30% strength gain with combined 8% cement + 6% zeolite). However, such studies often lack the statistical power to detect interactions, as highlighted by their marginal *p*-values. The conditional process model’s improved R^2^ (0.745 vs. 0.618 in moderation-only models) suggests that integrating mediation and moderation explains 12.7% more variance, a methodological advancement over isolated ANOVA or linear regression approaches [59].

The superiority of structural equation modeling (SEM) in capturing latent variables is apparent. For instance, Prado et al. [47] attribute a 15–20% CBR improvement to zeolite’s porosity effects but do not quantify mediation pathways. Similarly, zeolite-supported nanomaterials reduce As/Cd leaching by 40–60%, yet there is a lack of statistical frameworks to link physicochemical changes (e.g., surface area) to macro-scale outcomes [60]. While this study focuses on mechanical properties, literature like Khanmohammadi and Sadrara [59] highlights zeolite’s ion-exchange capacity for immobilizing Pb, Cd, and Zn. For example, He et al. [48] report a 70–90% metal retention using modified zeolite, mediated by surface area and cation exchange capacity (CEC). A parallel PCLR analysis could similarly elucidate the mediation pathways (e.g., CEC as a mediator between zeolite content and metal leaching), extending the methodological framework to environmental applications. The non-significant cement main effect (β = 1.874, *p* = 0.141) diverges from studies by Belviso [46], reporting 15–30% CBR increases with 5–10% cement. This discrepancy may stem from sample size limitations (power = 0.62 for β = 1.874) or contextual factors (e.g., soil type). Future work should integrate PCLR models with larger datasets to validate moderation effects. Additionally, extending the analysis to good water retention or hydraulic conductivity could unify the geotechnical applications of zeolite. The PCLR model provides a statistically rigorous framework for dissecting zeolite’s role in soil systems, revealing mediation effects (free swell index) and conditional interactions (cement–zeolite synergy) that traditional methods overlook. By quantitatively linking micro-scale mechanisms (e.g., ion exchange, porosity) to macro-scale outcomes (CBR, swelling), this approach offers a template for optimizing additives in soil stabilization and environmental remediation.

The scope of this study encompassed a high-plasticity clay (CH) soil from Kano State, Nigeria, stabilized with clinoptilolite zeolite and Type I/II cement. Laboratory tests evaluated swelling, shrinkage, and strength parameters under controlled conditions (22 °C), with statistical modeling via PCLR. Regarding limitations, the results are specific to the tested soil type and zeolite mineralogy, limiting direct extrapolation to other geologies. Field-scale validation and long-term durability under cyclic wetting–drying or freeze–thaw conditions were not assessed. Environmental impacts, such as leaching and the carbon footprint of zeolite–cement mixtures, require further analysis. For future research directions, it is recommended to expand testing to diverse soil types such as silts and sandy clays and various climatic conditions to generalize the findings. Investigation of the field performance of optimized mixtures (6% cement + 9–12% zeolite) in real infrastructure projects would provide practical validation. Assessment of the long-term chemical stability and leaching potential of stabilized soils is necessary for environmental safety evaluation. Exploration of hybrid additives combining zeolite with nano-silica or biochar could enhance their multifunctional properties, including strength, permeability, and contaminant adsorption. The development of machine learning models integrating field data would facilitate the optimization of admixture ratios for site-specific conditions.

## 4. Conclusions

In conclusion, this research provided valuable insights and a simplified data set representation. Based on the results, the following can be concluded:

1. The cement–zeolite combination demonstrated significant synergistic effects on expansive soil properties. With 6% cement and 12% zeolite, the FSI decreased from 45% to 16.5%, the swell percentage from 22.5% to 9%, and the swell pressure from 240 kPa to 110 kPa. The shrinkage percentage reduced from 5.6% to 2.8% with 9% zeolite and 6% cement, while the shrinkage coefficient decreased from 0.08 to 0.04005 with 6% cement and 12% zeolite. The bearing capacity improved substantially, with the unsoaked CBR increasing from 9.02% to 80.01% and the soaked CBR reaching 72.79% with 6% cement and 9% zeolite. This optimal blend showed only a 9.0% strength loss upon soaking, with a peak resilience ratio of 0.8010, indicating excellent moisture resistance;
2. Principal component analysis showed that PC1 accounted for 86.20% of the unsoaked CBR variance and 93.63% of the soaked CBR variance, representing key cement–zeolite synergistic interactions affecting the bearing capacity. The PCLR modeling approach demonstrated a strong predictive performance, with R-squared values of 0.935 for the unsoaked CBR and 0.750 for the soaked CBR, with RMSE values of 20% and 25%, respectively. This accuracy resulted from PCA’s dimensionality reduction combined with Lasso regression’s feature selection, effectively managing multicollinearity and identifying the most influential components of soil behavior;
3. Principal component analysis revealed that three principal components account for 95% of the total variance in soil properties. Mediation analysis established that zeolite content significantly affects the free swell index (path coefficient = −0.91429, *p* < 0.0001), with the free swell index significantly influencing the soaked CBR values (path coefficient = −2.13016, *p* < 0.0001). The total effect of zeolite content on the soaked CBR (coefficient = 1.894114, *p* = 0.000492) is statistically significant, while the direct effect (coefficient = −0.11108, *p* = 0.810543) is not, confirming that the relationship is fully mediated by the free swell index, with an indirect effect of 2.005192 (95% CI [1.144505, 3.052653]);
4. Moderation analysis demonstrated that zeolite content has a significant main effect on the soaked CBR (β = 1.3688, *p* = 0.050), with the overall moderation model explaining 61.8% of the variance in soaked CBR values. The conditional process model, incorporating both mediation and moderation mechanisms, exhibited enhanced explanatory power (R^2^ = 0.745), confirming the critical role of the free swell index as a mediating variable (β = −2.4083, *p* = 0.002). Although the interaction between zeolite and cement (β = 0.1428, *p* = 0.219) did not reach statistical significance at the conventional α = 0.05 level, graphical analysis suggests potential synergistic effects.

## Figures and Tables

**Table 1:** Key properties of materials: (A) soil, (B) zeolite, and (C) cement.

A. Natural Soil		
Category	Property	Value
Gradation	Gravel/Sand/Silt+Clay (%)	0.65/18.52/80.83
Particle Size	D_10_, D_30_, D_60_ (mm)	0.0055, 0.036, 0.065
Consistency	Liquid Limit/Plastic Limit/Plasticity Index (%)	68/19/49
Classification	USCS	CH
Compaction	Max. Dry Density (kN/m^3^)	17
	Optimum Moisture (%)	23.5
Strength	Unconfined Compressive Strength (UCS) (kPa)	409
Physical	Specific Gravity/pH	2.7/5.12
Mineralogy	Dominant Clay	Montmorillonite
Color		Gray
B. Zeolite Powder		
Category	Property	Value
Physical State		Powder
Density	Specific Gravity/Density (g/cm^3^)	2.36/2.46
Surface Properties	Cation Exchange Capacity (meq/g)/Surface Charge (meq/Å^2^)	1.1/11 × 10^−22^
	Total Surface Area (m^2^/g)	≤768
Porosity	Pore Volume (%)/Pore Size (Å)	≤49/5
pH		8.88
Color		White
C. Cement Powder		
Category	Property	Value
Setting Time	Initial/Final (min)	33.4/242
Physical	pH (aqueous solution)/Bulk Density (kg/m^3^)	13/1101
Strength	3-day UCS (MPa)	29.1
Quality	Fineness (m^2^/kg)/Soundness (mm)	362/0.38
Specific Gravity		3.13

**Table 2:** Chemical composition of the zeolite and cement.

Chemical Constituent	Composition of Cement (%)	Composition of Zeolite (%)
SiO_2_	19.3	58.9
Al_2_O_3_	3.67	11.3
Fe_2_O_3_	3.44	2.5
Na_2_O	0.26	3.8
K_2_O	0.78	3.7
CaO	62.62	0.6
TiO_2_	0.597	0.5
PbO	0	-
MgO	3.39	-
SO_3_	3.21	-
SrO_2_	-	-
P_2_O_5_	0.0897	-
NiO_2_	-	-
MnO	0.237	-
ZnO	-	-
CuO	-	-
Cr_2_O_3_	-	-
BaO	0	-
Cl	0.03	-
Loss on Ignition	2.38	19.03

**Table 3:** Results of the statistical analyses in zeolite–cement soil stabilization: (A) mediation, (B) moderation, and (C) conditional process analysis.

A. Mediation							
Property	Path	coef	std err	pval	CI [2.5%]	CI [97.5%]	sig
1	Free Swell Index ~ X	−0.91429	0.166446	7.22E-06	−1.25524	−0.57334	Yes
2	Y ~ Free Swell Index	−2.13016	0.246499	2.18E-09	−2.6351	−1.62523	Yes
3	Total	1.894114	0.480597	0.000492	0.909657	2.878572	Yes
4	Direct	−0.11108	0.458836	0.810543	−1.05253	0.830376	No
5	Indirect	2.005192	0.533386	0	1.144505	3.052653	Yes
B. Moderation							
Property		coef	std err	t	*p* > |t|	CI [2.5%]	CI [97.5%]
1	Intercept	19.5125	6.045	3.228	0.003	7.087	31.938
2	Q (“Zeolite Content (%)”)	1.3688	0.666	2.057	0.050	0.001	2.737
3	Q (“Cement Content (%)”)	1.8738	1.234	1.519	0.141	−0.662	4.410
4	Interaction	0.1313	0.136	0.967	0.343	−0.148	0.411
C. Conditional Process							
Property		coef	std err	t	*p* > |t|	CI [2.5%]	CI [97.5%]
1	Intercept	123.2297	29.813	4.133	0.000	61.828	184.632
2	Q (“Zeolite Content (%)”)	−0.8790	0.844	−1.041	0.308	−2.618	0.860
3	Q (“Cement Content (%)”)	−1.4634	1.397	−1.048	0.305	−4.340	1.413
4	Q (“Zeolite Content (%)”): Q (“Cement Content (%)”)	0.1428	0.113	1.261	0.219	−0.090	0.376
5	Q (“Free Swell Index”)	−2.4083	0.682	−3.530	0.002	−3.814	−1.003

## Data Availability

Some or all data, models, or codes that support the findings of this study are available from the corresponding author upon reasonable request.
